# Association between body mass index and reversion to normoglycemia from impaired fasting glucose among Chinese adults: a 5-year cohort study

**DOI:** 10.3389/fendo.2023.1111791

**Published:** 2023-04-18

**Authors:** Yong Han, Haofei Hu, Zhiqiang Huang, Dehong Liu

**Affiliations:** ^1^ Department of Emergency, Shenzhen Second People’s Hospital, Shenzhen, Guangdong, China; ^2^ Department of Nephrology, Shenzhen Second People’s Hospital, Shenzhen, Guangdong, China

**Keywords:** pre-diabetes, regression to normoglycemia, nonlinear, competitive risk model, smooth curve fitting

## Abstract

**Objective:**

Evidence regarding the relationship between body mass index (BMI) and reversion to normoglycemia from prediabetes is still limited. The purpose of our study is to survey the link of BMI on reversion to normoglycemia among patients with impaired fasting glucose (IFG).

**Methods:**

This study, a retrospective cohort, covered 32 regions and 11 cities in China and collected 258,74 IFG patients who underwent a health check from 2010 to 2016. We investigated the association between baseline BMI and reversion to normoglycemia in patients with IFG using the Cox proportional-hazards regression model. The nonlinear relationship between BMI and reversion to normoglycemia was determined using a Cox proportional hazards regression with cubic spline functions and smooth curve fitting. In addition, we also performed a series of sensitivity analyses and subgroup analyses. A competing risk multivariate Cox regression was performed using progression to diabetes as a competing risk for reversal of normoglycemic events.

**Results:**

After adjusting covariates, the results showed that BMI was negatively related to the probability of reversion to normoglycemia (HR=0.977, 95%CI:0.971-0.984). Compared with participants with normal BMI(<24kg/m^2^), overweight (BMI:24-28kg/m^2^) participants with IFG had a 9.9% lower probability of returning to normoglycemia (HR=0.901,95%CI:0.863-0.939), while obese patients (BMI ≥ 28kg/m^2^) had a 16.9% decreased probability of reverting from IFG to normoglycemia (HR=0.831,95%CI:0.780-0.886). There was also a nonlinear relationship between them, and the inflection point of BMI was 21.7kg/m^2^. The effect sizes (HR) on the left sides of the inflection point were 0.972(95%CI:0.964-0.980). The competing risks multivariate Cox’s regression and sensitivity analysis demonstrated the robustness of our results.

**Conclusion:**

This study demonstrates a negative and nonlinear relationship between BMI and reversion to normoglycemia in Chinese patients with IFG. Minimizing BMI to 21.7 kg/m^2^ in patients with IFG through aggressive intervention may significantly increase the probability of returning to normoglycemia.

## Introduction

Diabetes is a major public health concern because of its high prevalence, mortality, and rising costs (1). Prediabetes is an intermediate stage between normal glucose levels and type 2 diabetes mellitus (T2DM). It generally reflects the presence of either or both impaired fasting glucose (IFG) and glucose tolerance (IGT). In 2017, the International Diabetes Federation (IDF) estimated that 374 million adults worldwide had prediabetes, and the number of adults with prediabetes will reach 548 million by 2045, equaling 8.4% of the adult population (2). Approximately 86 million US adults (37%) have prediabetes (3). Among adults in China, the prevalence of prediabetes was about 35.7% (4). Adolescent boys and girls in India have a 12.3% prediabetes prevalence rate (5). Moreover, there is evidence that the prevalence of prediabetes in Korea is as high as 38.3% (6). People with prediabetes have an increased risk of T2DM, with approximately 5-10% of people developing T2DM each year, and up to 70% of them will eventually develop T2DM according to the American Diabetes Association (ADA) expert panel (3, 7). Nevertheless, it is worth noting that some patients with prediabetes do not progress to diabetes but remain in the prediabetic stage, and 20%-50% of individuals with prediabetes may even regress to normoglycemia (8–10). In addition, prediabetes increases the risk of not only T2DM but also cardiovascular disease and microvascular complications (11–13). Previous research has suggested that reversion to normoglycemia, even briefly, is related to a significantly decreased risk of development of T2DM in patients with prediabetes (14). Thus, the clinical benefits of reversion from prediabetes to normoglycemia cannot be overemphasized. The goal of prediabetes screening and treatment should be to revert normoglycemia.

Given that most of the attention on the clinical side seems to be focused on disease progression, finding contributing factors for prediabetes regression to normoglycemia is equally or more important to indicate pathways for prevention and actionable targets for sustaining public health efforts. Unfortunately, few studies have been conducted to determine the rate of reversion to normoglycemia in people with prediabetes and which contributing factors are associated with this. Preliminary evidence from previous epidemiological studies suggests that regression to normoglycemia is associated with factors such as age, baseline fasting glucose, insulin secretion, obesity, beta-cell function, fasting triglycerides, etc (3, 15–18). Studies have shown that an increase in body mass index (BMI) is positively associated with the risk of progression from prediabetes to diabetes (19, 20). However, there is limited research into the relationship between BMI and regression to normoglycemia from prediabetes. A cohort study revealed that an increase in delta-BMI (baseline BMI minus BMI at follow-up) was negatively associated with the likelihood of returning to normoglycemia in participants with prediabetes (21). Another study from Korea showed that in older adults, even modest weight loss helped to return from prediabetes to normoglycemia (22).

Regrettably, neither study performed subgroup analyses nor explored the non-linear relationship between BMI and regression to normoglycemia from prediabetes. Besides, the current study is limited by the small sample size. The link between BMI and reversion to normoglycemia has not yet been widely explored among Chinese adults. Furthermore, given that patients with diabetes at follow-up are no longer likely to regress from prediabetes to euglycemia, observation of the possibility of reversal of prediabetes to euglycemic events or the occurrence of altered events may be hampered. However, no research has attempted to investigate the relationship between them using the competing risk model. Therefore, based on the fact that obesity is a high-risk factor for diabetes, we propose the hypothesis that there may be a negative association between BMI and the likelihood of reversal of prediabetes to normoglycemia in the Chinese population, and that a non-linear relationship between them cannot be excluded. We conducted a retrospective cohort study using published Chinese population-based data to test this hypothesis.

## Methods

### Study design

This study used a retrospective cohort study design, and the data were obtained from a retrospective cohort study previously undertaken by Chinese researchers (Chen et al.) from a computerized database in China (23). The target-independent variable was BMI at baseline. The outcome variable was reversion to normoglycemia from prediabetes at follow-up.

### Data source

The raw data were obtained free of charge from DATADRYAD (www.datadryad.org) and provided by Ying Chen et al. With reference to the terms of service of the Dryad database, the dataset can be used by researchers to be able to share, remix, modify and create derivative works for non-commercial purposes, as long as the author and source are credited. Data information was obtained from an open access article published in 2018 – “Association of body mass index and age with diabetes onset in Chinese adults: a population-based cohort study” (http://dx.doi.org/10.1136/bmjopen-2018-021768). The data can be downloaded at: https://doi.org/10.5061/dryad.ft8750v (23).

### Study population

The initial researchers took information from a computerized database created by the Rich Healthcare Group in China. This database contains all medical records of participants who underwent a health check from 2010 to 2016, spanning 32 regions and 11 cities in China. The Rich Healthcare Group Review Board initially approved the original study, and the information was retrieved retrospectively. For the retrospective study, no informed consent or approval was required by the institutional ethics committee (23). Therefore, the current secondary analysis did not require ethical approval. Additionally, the initial study was conducted in accordance with the Helsinki Declaration (23). So did this secondary analysis.

**Figure 1:**
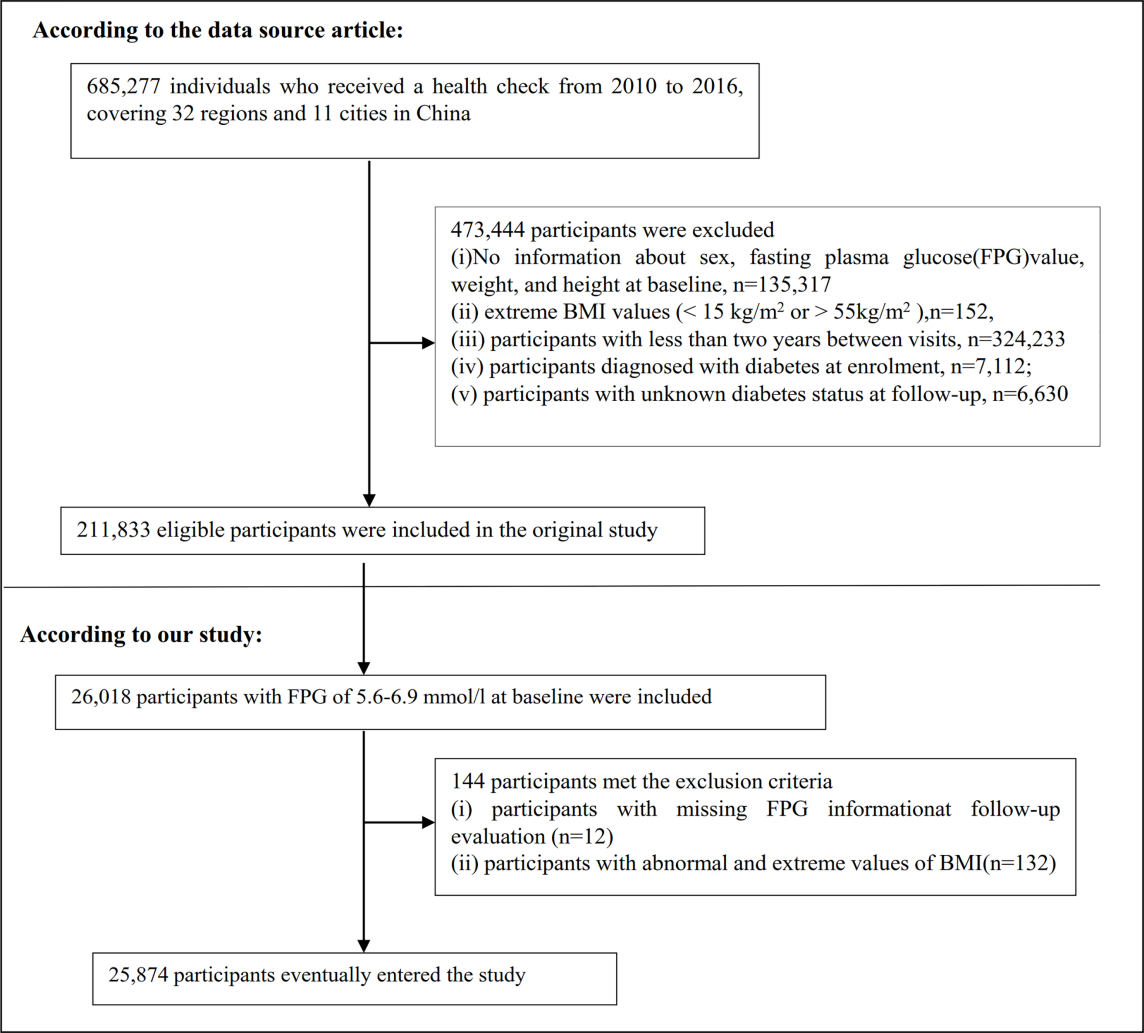
Flowchart of study participants.

The original study enrolled 685,277 participants older than 20 who passed at least two health examinations. 473,444 participants meeting the exclusion criteria were excluded. The following were the original study’s exclusion criteria: The original study’s exclusion criteria were as follows: (i) participants with a visit interval of less than two years; (ii) participants with extreme BMI values (15 kg/m^2^ or > 55 kg/m^2^); (iii) participants with no information about weight, height, sex, and fasting plasma glucose(FPG) value at baseline; (iv) participants with diabetes at enrollment; and (v) participants whose diabetes status at follow-up was unknown. Finally, the analysis of the initial study comprised a total of 211,833 people (23). In the current study, we first further included 26,018 participants with baseline FPG of 5.6-6. 9 mmol/L. We then excluded participants with missing FPG information at follow-up (n = 12) as well as those with abnormal and extreme BMI (three standard deviations greater or less than three standard deviations from the mean) (n = 132). Finally, the current study included 25,874 people in total. The procedure for choosing participants is shown in **Figure 1**. It is important to highlight that according to the American Diabetes Association 2022 criteria, prediabetes is defined as the presence of IFG (FPG level of 5.6–6.9 mmol) and/or IGT and/or hemoglobin A1c(HA1c) (24). Our definition of prediabetes is therefore based on FPG. To make the study more accurate, our study population is reported as patients with IFG.

### Variables

#### Body mass index

BMI was recorded as a continuous variable. The detailed procedure for defining BMI was as follows: BMI = weight/height^2^(kg/m^2^). It was important to note that relevant information for height and weight was obtained at baseline. The categories of obesity (BMI≥ 28 kg/m^2^), overweight (24≤ BMI < 28 kg/m^2^), and normal weight (BMI< 24 kg/m^2^) were established according to the definition put forth by the Working Group on Obesity in China (25).

#### Outcome measures

The occurrences of reversion to normoglycemia were our intriguing outcome variable. Reversion to normoglycemia was based on FPG<5.6mmol/l at follow-up evaluation and the absence of self-reported incident diabetes (26, 27).

#### Covariates

The covariates in our study were selected based on the original study, previous studies having a correlation to diabetes or prediabetes, and our clinical expertise (18, 22, 23, 28, 29). Covariates included the following variables: (i)categorical variables: sex, smoking status, family history of diabetes, and drinking status. (ii) continuous variables: weight, height, age, serum creatinine (Scr), systolic blood pressure (SBP), aspartate aminotransferase (AST), triglyceride (TG), high-density lipoprotein cholesterol (HDL-c), diastolic blood pressure (DBP), alanine aminotransferase (ALT), total cholesterol (TC), blood urea nitrogen (BUN), low-density lipid cholesterol (LDL-c).

#### Data collection

In the original study, professional researchers used standard questionnaires to gather baseline data on drinking, smoking, and family history of diabetes. Standard mercury sphygmomanometers measured blood pressure. During each visit, fasting venous blood samples were taken at least 10 hours after a fast. A Beckman 5800 autoanalyzer was used to measure plasma glucose, HDL-c, TC, LDL-c, BUN, TG, AST, ALT, and Scr. The time to regression to normoglycemia or progression to diabetes was based on when participants returned for one or more physical examinations.

#### Missing data processing

In current study, the number of participants whose data are missing of DBP, SBP, TC, ALT, TG, BUN, Scr, LDL-c, HDL-c, AST, drinking status, and smoking status was 7(0.03%), 7(0.03%), 605(2.34%),232(0.90%), 607(2.35%),2840(10.98%), 1334(5.16%), 9897(38.25%), 10527(40.69%), 14629(56.54%), 17139(66.24%), and 17139(66.24%), respectively. This study used multiple imputations for missing data to reduce the volatility brought on by missing variables. SBP, age, ALT, sex, LDL-c, DBP, AST, TG, Scr, HDL-c, BUN, TC, drinking status, family history of diabetes, and smoking status were all included in the imputation model (iterations were 10; the type of regression was linear). Missing-at-random (MAR) assumptions are used in missing data analysis processes (30, 31).

### Statistical analysis

We divided the individuals into three categories based on the World Health Organization’s BMI values for Chinese patients: “normal”, “overweight”, and “obesity”. The means and standard deviations were presented for continuous variables with Gaussian distributions, medians were reported for skewed distributions, and frequencies and percentages were presented for categorical variables. We used the Kruskal-Wallis H test (skewed distribution), the One-Way ANOVA test (normal distribution), or χ2 (categorical variables) to test for differences among different BMI groups.

Following collinearity screening, we used univariate and multivariate Cox proportional-hazards regression models to examine the relationship between BMI and the reversion rate to normoglycemia in individuals with IFG, including a crude model with no covariates adjusted, a model with just minimal covariates adjusted (Model I with adjusted sex and age), and a model with full covariate adjustments (Model II: adjusted DBP, age, sex, SBP, AST, BUN, ALT, LDL-c, Scr, TG, HDL-c, family history of diabetes, drinking status, and smoking status). Effect sizes (HR) with 95% confidence intervals (CI) were recorded. We adjusted for confounding factors based on clinical experience, literature reports, and the results of univariate analysis. Additionally, the final multivariate Cox proportional hazards regression equation did not include TC since it was collinear with other variables (**Supplementary Table S1**).

Besides, the Cox proportional hazards regression model with cubic spline functions and smooth curve fitting were performed to account for the nonlinear relationship between BMI and reversion to normoglycemia in participants with IFG. Furthermore, a two-piecewise Cox proportional hazards regression model was used to clarify the nonlinear association between BMI and reversion from IFG to normoglycemia. Finally, a log-likelihood ratio test was performed to choose the best model to explain the association between them in patients with IFG. Considering that patients who experience diabetes at follow-up are no longer likely to recover from IFG to normoglycemia, this may hinder the observation of prediabetes reversal to normoglycemia events or alter the likelihood of events occurring (32, 33). Therefore, competing risks multivariate Cox proportional-hazards regression was performed, as described by Fine and Gray, with progression to diabetes as the competing risk for the reversal to normoglycemia events (33, 34).

Using a stratified Cox proportional hazard regression model, subgroup analyses were performed across various groupings (age, sex, SBP, DBP, smoking status, and drinking status). First, based on clinical cut-off points, continuous data, such as SBP and age, were transformed into categorical variables (age: 30, 30 to 40, 40 to 50, 50 to 60, 60 to 70, 70 years old; SBP: 140, 140 mmHg) (35). In addition to the stratification factor itself, we adjusted each stratification for DBP, age, SBP, sex, Scr, ALT, HDL-c, AST, BUN, TG, LDL-c, drinking status, family history of diabetes, and smoking status. Ultimately, in models with and without interaction terms, the likelihood ratio test was employed to identify whether there were interaction terms or not.

To check the reliability of the findings, we ran a series of sensitivity analyses. Previous studies have suggested that drinking status, TG, and family history of diabetes are significantly related to glucose metabolism (36–38). We also conducted further sensitivity analyses to examine the connection between BMI and reversion to normoglycemia in prediabetic patients. Firstly, we performed a sensitivity analysis on participants who had never consumed alcohol (n=21,010). We also performed a sensitivity analysis after excluding patients with a family history of diabetes (n=25,244). In addition, we further explored the relationship between BMI and reversion to normoglycemia in participants with TG<1.7mmol/L (N= 15,858). The continuity covariate was also incorporated into the equation as a curve using a generalized additive model (GAM) to confirm the reliability of the findings. We also calculated E-values to examine the possibility of unmeasured confounding between BMI and reversion from IFG to normoglycemia (39).

All results were written in accordance with the STROBE statement (40). Empower Stats (X&Y Solutions, Inc., Boston, MA, http://www.empowerstats.com) and R statistical software packages (http://www.r-project.org, The R Foundation) were used for all analyses. Statistical significance was set at P values lower than 0.05(two-sided). **Supplementary Figure S1** showed the analytical framework for this study.

## Results

### Characteristics of participants

**Figure 2:**
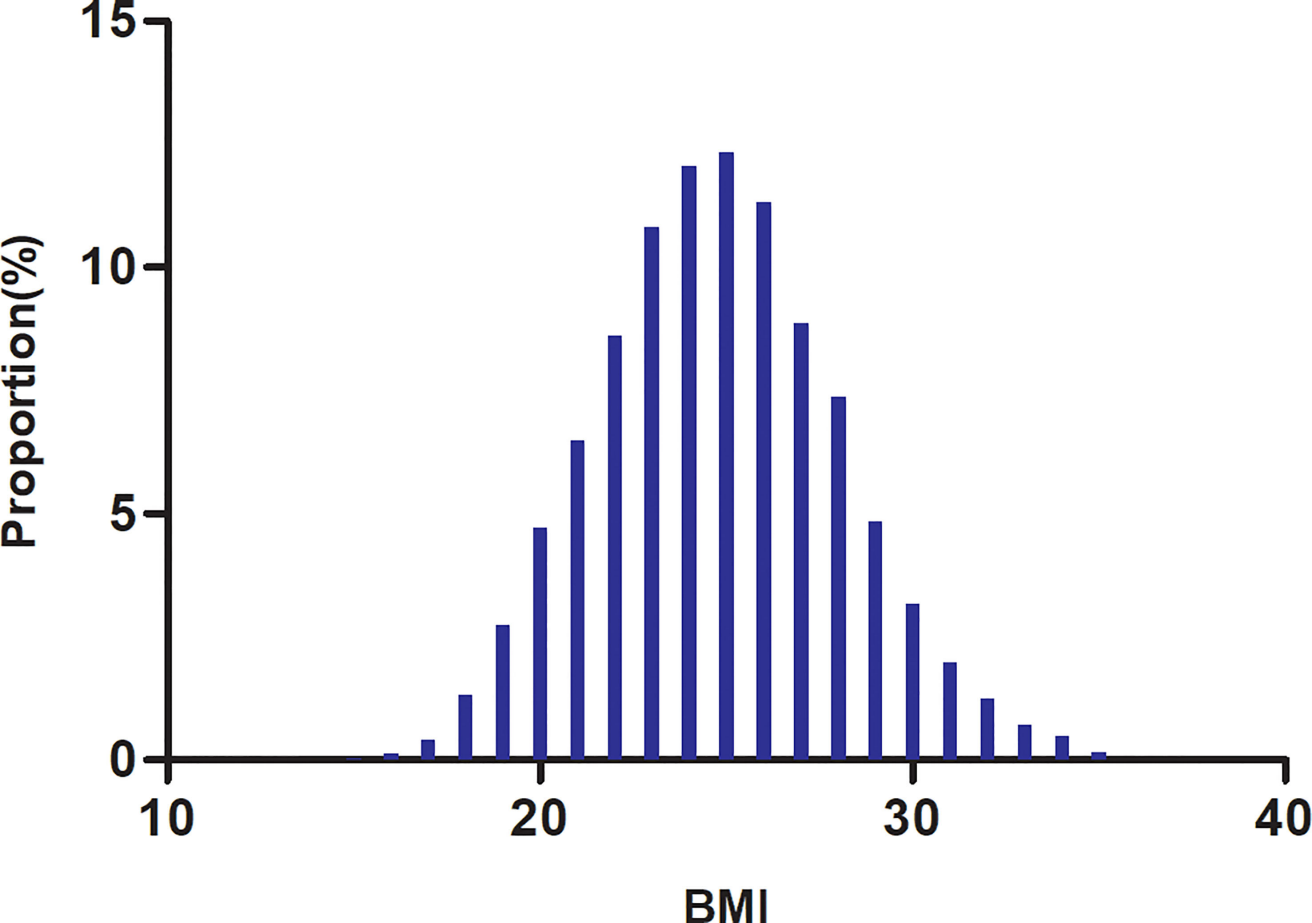
Distribution of BMI. It presented a normal distribution, ranging from 15.2 to 34.9kg/m^2^, with a mean of 24.74kg/m^2^.

The study participants’ demographic and clinical characteristics are presented in **Table 1**. The mean age was 49.07 ± 13.82 years old, and 17,168 (66.35%) were male. The median follow-up time was 3.05 years, and 11,856 (45.82%) participants had a final reversion to normoglycemia. BMI presents a normal distribution, ranging from 15.2 to 34.9kg/m^2^, with a mean of 24.74kg/m^2^ (**Figure 2**). We assigned adults into subgroups based on Chinese criteria for BMI categories (normal: < 24, overweight: 24-28, obesity: ≥28kg/m^2^). Compared with the normal group, age, height, weight, DBP, SBP, TG, LDL-c, TC, AST, ALT, Scr, and BUN increased significantly in the obesity group, whereas the opposite results were found in the HDL-c covariates. In addition, the proportion of men, current smokers, and current drinkers was higher in the obesity group.

**Table 1:** The baseline characteristics of participants.

BMI groups (kg/m^2^)	normal (<24)	overweight (24-28)	obesity (≥28)	P-value
participants	10688	11014	4172	
Sex				<0.001
Male	5892 (55.13%)	8107 (73.61%)	3169 (75.96%)	
Female	4796 (44.87%)	2907 (26.39%)	1003 (24.04%)	
SBP (mmHg)	123.15 ± 17.18	128.62 ± 17.05	133.33 ± 17.34	<0.001
DBP (mmHg)	75.42 ± 10.47	79.58 ± 10.87	82.75 ± 11.40	<0.001
Age(years)	47.40 ± 14.37	50.68 ± 13.19	49.12 ± 13.50	<0.001
Height(cm)	165.73 ± 8.39	167.22 ± 8.17	167.80 ± 8.36	<0.001
Weight(kg)	59.79 ± 7.84	72.24 ± 7.78	84.26 ± 9.60	<0.001
BMI (kg/m^2^)	21.69 ± 1.67	25.77 ± 1.12	29.84 ± 1.59	<0.001
AST(U/L)	23.78 ± 9.56	27.14 ± 11.72	31.25 ± 15.72	<0.001
HDL-c(mmol/L)	1.40 ± 0.31	1.30 ± 0.28	1.24 ± 0.29	<0.001
TC (mmol/L)	4.85 ± 0.95	5.04 ± 0.95	5.12 ± 0.97	<0.001
TG (mmol/L)	1.39 ± 1.11	1.98 ± 1.58	2.28 ± 1.60	<0.001
LDL-c(mmol/L)	2.81 ± 0.72	2.92 ± 0.71	2.97 ± 0.74	<0.001
ALT(U/L)	17.20 (13.00-24.30)	24.40 (17.70-35.90	32.00 (22.00-50.00)	<0.001
Scr (μmol/L)	70.05 ± 16.13	74.56 ± 15.67	75.10 ± 15.75	<0.001
BUN (mmol/L)	4.86 ± 1.24	5.08 ± 1.24	5.07 ± 1.25	<0.001
Drinking status				<0.001
Current drinker	257 (2.40%)	490 (4.45%)	218 (5.23%)	
Ever drinker	1200 (11.23%)	1879 (17.06%)	820 (19.65%)	
Never	9231 (86.37%)	8645 (78.49%)	3134 (75.12%)	
Smoking status				<0.001
Current smoker	1796 (16.80%)	2896 (26.29%)	1210 (29.00%)	
Ever smoker	379 (3.55%)	537 (4.88%)	214 (5.13%)	
Never	8513 (79.65%)	7581 (68.83%)	2748 (65.87%)	
Family history of diabetes				0.342
No	10419 (97.48%)	10763 (97.72%)	4062 (97.36%)	
Yes	269 (2.52%)	251 (2.28%)	110 (2.64%)	
Follow up-times(years)	2.90 (2.09-3.77)	2.78 (2.10-3.15)	3.03 (2.26-3.98)	<0.001

Continuous variables were summarized as mean (SD) or medians (quartile interval); categorical variables were displayed as a percentage (%).

DBP, diastolic blood pressure; BMI, body mass index; TC, total cholesterol, SBP, systolic blood pressure; TG triglyceride, BMI, body mass index; AST aspartate aminotransferase; LDL-c, low-density lipid cholesterol; ALT, alanine aminotransferase; BUN, blood urea nitrogen; HDL-c, high-density lipoprotein cholesterol; Scr, serum creatinine.

Baseline characteristics according to regression and progression status of patients with IFG are shown in **Supplementary Table S2**. Participants who progressed to diabetes had significantly higher levels of age, height, weight, BMI, DBP, SBP, TG, LDL-c, TC, AST, ALT, Scr, and BUN than participants with persistent IFG but significantly lower levels of HDL-c. Besides, compared with participants with persistent IFG, age, height, weight, BMI, DBP, SBP, TG, LDL-c, TC, AST, ALT, Scr, and BUN decreased significantly in participants who reverted to normoglycemia, whereas the opposite results were found in the HDL-c covariates.

### The reversal rate to normoglycemia from IFG

**Figure 3:**
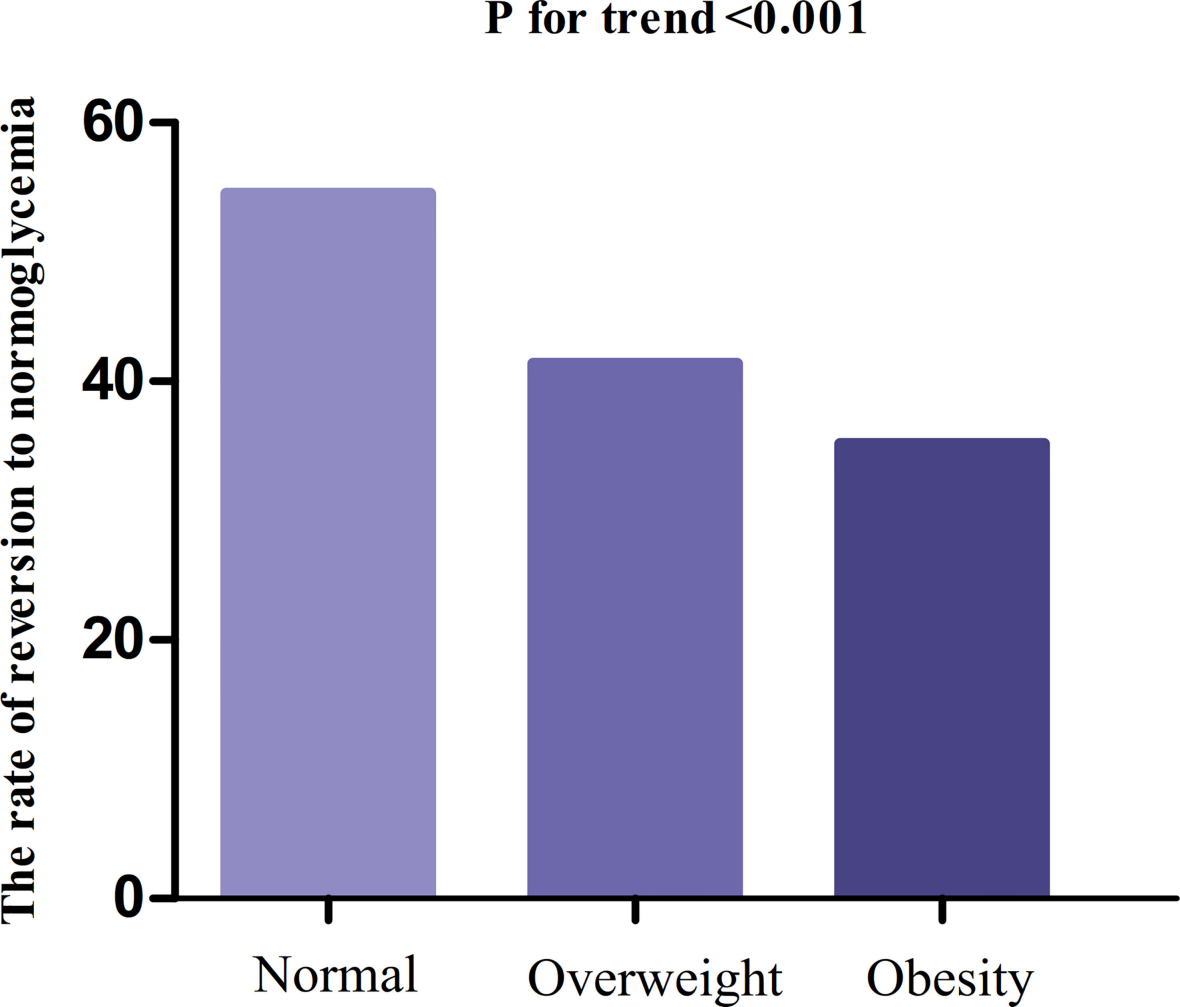
The rate of reversion to normoglycemia in people with IFG stratified by BMI. Participants with higher BMI had a significantly lower reversal rate than those with a lower BMI (p<0.001 for trend).

In participants with IFG, 11,856 individuals developed diabetes. The overall rate of reversion to normoglycemia was 155.33 per 1000 person-years. In particular, the reversal rate to normoglycemia among participants with IFG of BMI groups was normal group:186.84, overweight group:139.14, and obesity group:119.25 per 1000 person-years, respectively. The overall cumulative reversal rate of IFG to normoglycemia was 45.82% over a median follow-up period of 3.05 years. The cumulative reversal rate in each BMI group was normal group:54.55%, overweight group: 41.38%, and obesity group: 35.19% (**Figure 3**). Participants with higher BMI had a significantly lower reversal rate than those with a lower BMI (p<0.001 for trend) (**Table 2**, **Figure 3**).

**Table 2:** The rate of reversion to normoglycemia in people with IFG (% or Per 1000 person-year).

BMI Group	Participants(n)		Reversion events(n)	Reversal rate (95% CI) (%)	Per 1000 person-year
**Total**	25874		11856	45.82(45.21-46.23)	155.33
**Normal**	10688		5830	54.55(53.60-55.49)	186.84
**Overweight**	11041		4558	41.38(40.46-42.30)	139.14
**Obesity**	4172		1468	35.19(33.74-36.64)	119.25
**P for trend **				<0.001	

**Figure 4:**
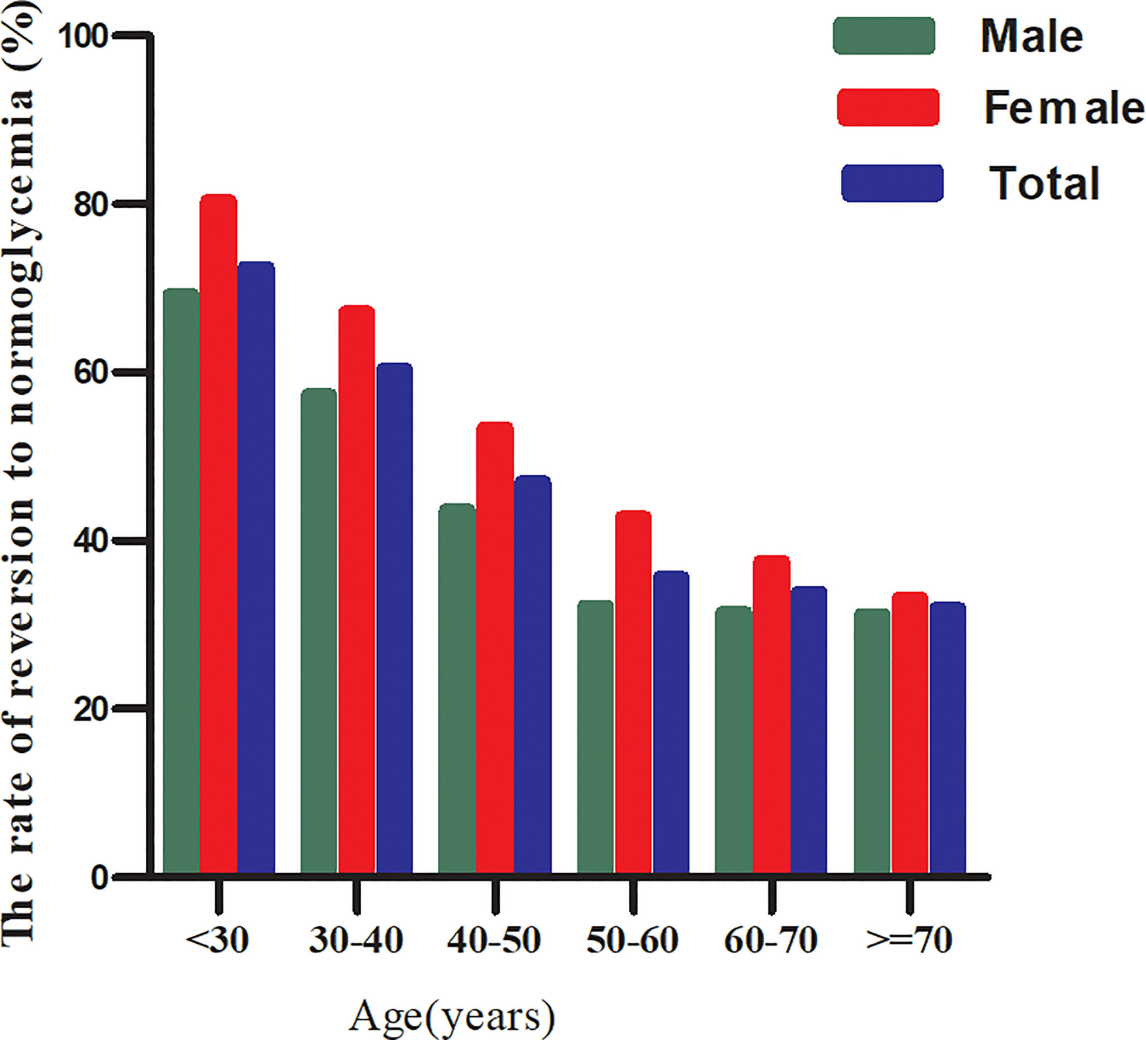
The rate of reversion to normoglycemia in IFG patients of age stratification by 10 intervals. Figure showed that the rate of reversion to normoglycemia among participants with IFG was higher in women than in men, regardless of their age group. It was also found that the reversal rate decreased with age in both men and women.

BMI, body mass index; CI, confidence interval,

In the age stratification by ten intervals, the rate of reversion to normoglycemia among participants with IFG was higher in women than in men, regardless of their age group (**Figure 4**). It was also found that the reversal rate decreased with age in both men and women.

### Factors influencing reversion to normoglycemia among participants with IFG analyzed by univariate Cox proportional hazards regression

Univariate analyses showed that reversion to normoglycemia in patients with IFG was negatively correlated with age, BMI, DBP, SBP, ALT, AST, TG, TC, LDL-c, BUN, and family history of diabetes but was positively related to HDL-c, never smoking and never drinking (all P<0.05; **Table 3**).

**Table 3:** Factors influencing reversion to normoglycemia among participants with IFG analyzed by univariate Cox proportional hazards regression.

Variable	Characteristics	HR (95% CI)	P-value
Age (years)	49.071 ± 13.818	0.976 (0.975, 0.978)	<0.001
Sex			
Male	17168 (66.352%)	Ref	
Female	8706 (33.648%)	1.267 (1.221, 1.315)	<0.001
BMI (kg/m^2^)	24.742 ± 3.251	0.935 (0.930, 0.940)	<0.001
SBP (mmHg)	127.120 ± 17.546	0.990 (0.988, 0.991)	<0.001
DBP (mmHg)	78.371 ± 11.129	0.985 (0.983, 0.987)	<0.001
TC (mmol/L)	4.972 ± 0.957	0.880 (0.863, 0.898)	<0.001
TG (mmol/L)	1.787 ± 1.450	0.891 (0.877, 0.905)	<0.001
HDL-c(mmol/L)	1.331 ± 0.304	1.612 (1.523, 1.706)	<0.001
LDL-c(mmol/L)	2.882 ± 0.722	0.919 (0.896, 0.943)	<0.001
ALT (U/L)	28.363 ± 23.334	0.993 (0.992, 0.994)	<0.001
AST (U/L)	26.413 ± 11.954	0.987 (0.985, 0.989)	<0.001
BUN (mmol/L)	4.986 ± 1.248	0.955 (0.941, 0.969)	<0.001
Scr (μmol/L)	72.784 ± 16.039	0.997 (0.996, 0.998)	<0.001
Smoking status			
Current smoker	5902 (22.811%)	Ref	<0.001
Ever smoker	1130 (4.367%)	1.062 (0.963, 1.171) 0.227	<0.001
Never	18842 (72.822%)	1.255 (1.200, 1.313)	<0.001
Drinking status			
Current drinker	965 (3.730%)	Ref	<0.001
Ever drinker	3899 (15.069%)	1.260 (1.123, 1.413)	<0.001
Never	21010 (81.201%)	1.394 (1.253, 1.551)	<0.001
Family history of diabetes			
No	25244 (97.565%)	Ref	
Yes	630 (2.435%)	0.753 (0.666, 0.852)	< 0.001

Continuous variables were summarized as mean (SD) or medians (quartile interval); categorical variables were displayed as percentage (%).

DBP, diastolic blood pressure; BMI, body mass index; TC, total cholesterol, SBP, systolic blood pressure; TG triglyceride, BMI, body mass index; AST aspartate aminotransferase; LDL-c, low-density lipid cholesterol; ALT, alanine aminotransferase; BUN, blood urea nitrogen; HDL-c, high-density lipoprotein cholesterol; Scr, serum creatinine.

**Figure 5:**
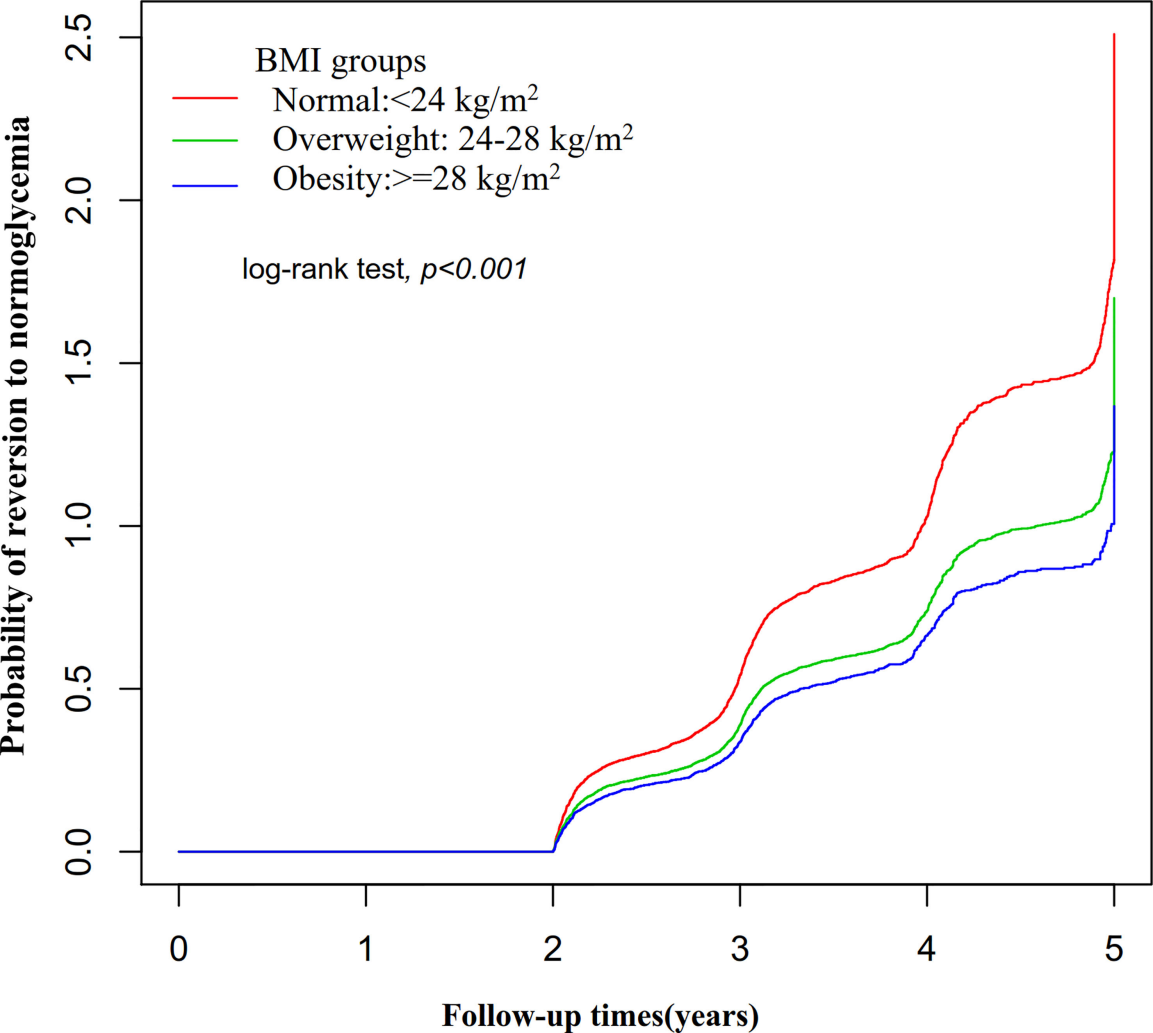
Kaplan-Meier curves for the probability of reversion to normoglycemia from IFG. Figure showed the Kaplan-Meier curves for the probability of reversion to normoglycemia from IFG stratified by BMI category. The probability of reversion to normoglycemia decreased progressively with rising BMI, meaning that Patients with the highest BMI had the lowest probability of reverting from IFG to normoglycemia.

**Figure 5** showed the Kaplan-Meier curves for the probability of reversion to normoglycemia from IFG stratified by BMI category. The probability of reversal to normoglycemia from IFG varied significantly between BMI groups (log-rank test, p<0.001). The probability of reversion to normoglycemia decreased progressively with rising BMI, meaning that patients with the highest BMI had the lowest probability of reverting from IFG to normoglycemia. **Supplementary Figure S2** presented Kaplan-Meier survival curves for diabetes-free survival probability. Among BMI groups, there were statistically significant differences in the probability of diabetes-free survival (log-rank test, p<0.001). IFG patients with the greatest BMI had the highest risk of progression to diabetes.

### The relationship between BMI and reversion to normoglycemia from IFG analyzed by multivariate Cox proportional-hazards regression model

We constructed three models using the Cox proportional-hazards regression model to investigate the association between BMI and reversion to normoglycemia in patients with IFG. In the crude model, a 1kg/m^2^ increase in BMI was associated with a 6.5% decrease in the probability of reversion to normoglycemia (HR=0.935,95%CI 0.930-0.940, p<0.001). In the minimally-adjusted model, when we adjusted for population variables only, each 1kg/m^2^ increase in BMI was associated with a 4.6% lower probability of reversion to normoglycemia (HR=0.954, 95%CI 0.949-0.960, p<0.001). The HR between BMI and reversion to normoglycemia from IFG was 0.977 (95% CI: 0.971-0.984, p<0.001) in the fully adjusted model. The distribution of confidence intervals suggested that the link between BMI and reversion to normoglycemia among patients with IFG obtained by the model was reliable (**Table 4**).

**Table 4:** Relationship between BMI and reversion to normoglycemia in patients with IFG in different models.

Exposure	Crude model (HR,95%CI)	Model I(HR,95%CI) P	Model II(HR,95%CI) P	Model III(HR,95%CI) P
BMI (kg/m^2^)	0.935 (0.930, 0.940) <0.001	0.954 (0.949, 0.960) <0.001	0.977 (0.971, 0.984) <0.001	0.982 (0.976, 0.989) <0.001
BMI Group				
Normal	Ref	Ref	Ref	Ref
Overweight	0.715 (0.688, 0.743) <0.001	0.809 (0.777, 0.842) <0.001	0.901 (0.863, 0.939) <0.001	0.928 (0.888, 0.969) <0.001
Obesity	0.618 (0.584, 0.654) <0.001	0.678 (0.640, 0.719) <0.001	0.831 (0.780, 0.886) <0.001	0.855 (0.802, 0.912) <0.001
P for trend	<0.001	<0.001	<0.001	<0.001

Crude model: we did not adjust other covariates.

Model I: we adjusted age, sex.

Model II: we adjusted age, sex, SBP, DBP ALT, AST, BUN, Scr, TG, LDL-c, HDL-c, family history of diabetes, drinking status, and smoking status.

Model III: we adjusted age(smooth), sex, SBP (smooth), DBP (smooth), Scr(smooth), TG (smooth), ALT(smooth), AST(smooth), LDL-c(smooth), HDL-c(smooth), smoking status, drinking status, family history of diabetes.

HR, Hazard ratios; CI, confidence, Ref, reference.

Besides, we transformed BMI from a continuous variable to a categorical variable and then reintroduced the categorically transformed BMI into the model. The results of the multivariate-adjusted model showed that with reference to participants with normal BMI, the HR was 0.901(95%CI:0.863-0.939) for overweight participants and 0.831(95%CI:0.780-0.886) for obese participants. That is, compared with participants with normal BMI(<24kg/m^2^), overweight participants (BMI:24-28kg/m^2^) with prediabetes had a 9.9% lower probability of returning to normoglycemia, while obese patients (BMI ≥ 28kg/m^2^) had a 16.9% decreased probability of reverting from IFG to normoglycemia (**Table 4** Model II).

### The results of competing risks multivariate Cox proportional-hazards regression

When progression to incident diabetes from IFG was treated as a competing event, the competing analysis results were shown in **Table 5**. In the crude model, BMI was negatively related to the probability of reversion to normoglycemia (SHR=0.93, 95% CI:0.93-0.94). In the minimally adjusted model (model I: adjusted age, sex), the result did not have a noticeable change (SHR:0.95, 95%CI: 0.95-0.96, p<0.001). In the fully adjusted model (model II) (adjusted age, sex, SBP, DBP ALT, AST, BUN, Scr, TG, LDL-c, HDL-c, family history of diabetes, drinking status, and smoking status), we could also detect a negative association between BMI and reversion to normoglycemia (SHR=0.92, 95%CI: 0.89-0.96). In addition, when BMI was used as a categorical variable, multivariate-adjusted model (fully adjusted model) results showed that overweight participants with IFG had a 10% lower probability of returning to normoglycemia compared with participants with normal BMI (SHR=0.90, 95%CI: 0.86-0.94), while obese patients had a 17.0% decreased probability of reverting from IFG to normoglycemia compared with patients with normal BMI (SHR=0.83, 95%CI: 0.78-0.89).

**Table 5:** Relationship between BMI and reversion to normoglycemia in patients with IFG in different models with competing risk of progression to diabetes.

Exposure	Crude model (SHR,95%CI, P)	Model I(SHR,95%CI, P)	Model II (SHR,95%CI, P)
BMI (kg/m^2^)	0.93 (0.93, 0.94) <0.001	0.95 (0.95, 0.96) <0.001	0.98 (0.97, 0.98) <0.001
BMI Group			
Normal	Ref.	Ref.	Ref.
Overweight	0.77 (0.74, 0.81) <0.001	0.81 (0.78, 0.84) <0.001	0.90 (0.86, 0.94) <0.001
Obesity	0.66 (0.63, 0.69) <0.001	0.68 (0.64, 0.72) <0.001	0.83 (0.78, 0.89) <0.001
P for trend	<0.001	<0.001	<0.001

Crude model: we did not adjust other covariates.

Model I: we adjust age, sex,

Model II: we adjust age, sex, SBP, DBP ALT, AST, BUN, Scr, TG, LDL-c, HDL-c, family history of diabetes, drinking status, and smoking status.

SHR, subdistribution hazard ratios; CI, confidence, Ref, reference

### Sensitivity analysis

A series of sensitivity analyses were performed to ensure that our findings were robust. We first introduced the continuity covariate as a curve into the equation using a GAM. As shown in **Table 4**, the outcome of Model III was consistent with the fully adjusted model. Referring to patients with IFG with normal BMI, obese patients had a 14.5% lower probability of reverting to normoglycemia (HR = 0.855, 95% CI: 0.802-0.912).

Furthermore, we conducted a sensitivity analysis on participants who had never consumed alcohol (n = 21,010). After adjusting for confounding variables (including DBP, age, SBP, sex, TG, ALT, AST, LDL-c, BUN, Scr, HDL-c, family history of diabetes, and smoking status), the findings indicated that BMI was also negatively linked with reversion to normoglycemia from IFG (HR=0.979, 95%CI:0.972-0.986, p<0.001). We also excluded patients with a family history of diabetes for the sensitivity analyses (n=25,244). After adjusting for confounding variables (including BUN, sex, AST, SBP, age, DBP, HDL-c, ALT, Scr, LDL-c, TG, drinking status, and smoking status), the results suggested that BMI was still negatively associated with reversion to normoglycemia from IFG (HR=0.977, 95% CI:0.971-0.984, p<0.001). Besides restricting the analysis to participants with TG<1.7mmol/L (adjusted for age, sex, SBP, DBP, ALT, AST, BUN, Scr, LDL-c, HDL-c, family history of diabetes, drinking status, and smoking status), the results suggested that the HR between BMI and probability of reverting to normoglycemia was 0.981 (95% CI:0.973-0.989, P<0.001). Similarly, when BMI was used as a categorical variable, sensitivity analyses of multivariate-adjusted models showed a significantly lower probability of recovery from IFG to normoglycemia in overweight and obese patients compared with participants with normal BMI (**Table 6**). Based on all the sensitivity analyses, it is evident that our findings were robust. We also calculated an E-value to evaluate the sensitivity to unmeasured confounding. Unknown or unmeasured variables likely had little impact on the association between BMI and recovery from IFG to normoglycemia, as the E-value (1.53) was greater than the relative risk of BMI and unmeasured confounders (1.36).

**Table 6:** Relationship between BMI and the probability of reverting from IFG to normoglycemia in different sensitivity analyses.

Exposure	Model I(HR,95%CI) P	Model II(HR,95%CI) P	Model III(HR,95%CI) P
BMI (kg/m^2^)	0.981 (0.973, 0.989) <0.00001	0.977 (0.971, 0.984) <0.00001	0.979 (0.972, 0.986) <0.00001
BMI Group			
Normal	Ref	Ref	Ref
Overweight	0.900 (0.855, 0.947) 0.00005	0.901 (0.863, 0.940) <0.00001	0.911 (0.869, 0.954) 0.00008
Obesity	0.875 (0.803, 0.955) 0.00267	0.832 (0.780, 0.887) <0.00001	0.849 (0.791, 0.912) <0.00001
P for trend	0.921 (0.887, 0.957) 0.00002	0.909 (0.882, 0.937) <0.00001	0.918 (0.889, 0.949) <0.00001

Model I was a sensitivity analysis performed after excluding participants with TG≥1.7 mmol/L (N= 15858). We adjusted age, sex, SBP, DBP, ALT, AST, BUN, Scr, LDL-c, HDL-c, family history of diabetes, drinking status, and smoking status.

Model II was a sensitivity analysis performed on participants without a family history of diabetes (N= 25244). We adjusted age, sex, SBP, DBP, ALT, AST, BUN, Scr, TG, LDL-c, HDL-c, drinking status, and smoking status.

Model III was a sensitivity analysis performed on participants who had never consumed alcohol (N= 21010). We adjusted age, sex, SBP, DBP, ALT, AST, BUN, Scr, TG, LDL-c, HDL-c, family history of diabetes, and smoking status.

HR, Hazard ratios; CI, confidence, Ref, reference.

### Cox proportional hazards regression model with cubic spline functions to address nonlinearity

**Figure 6:**
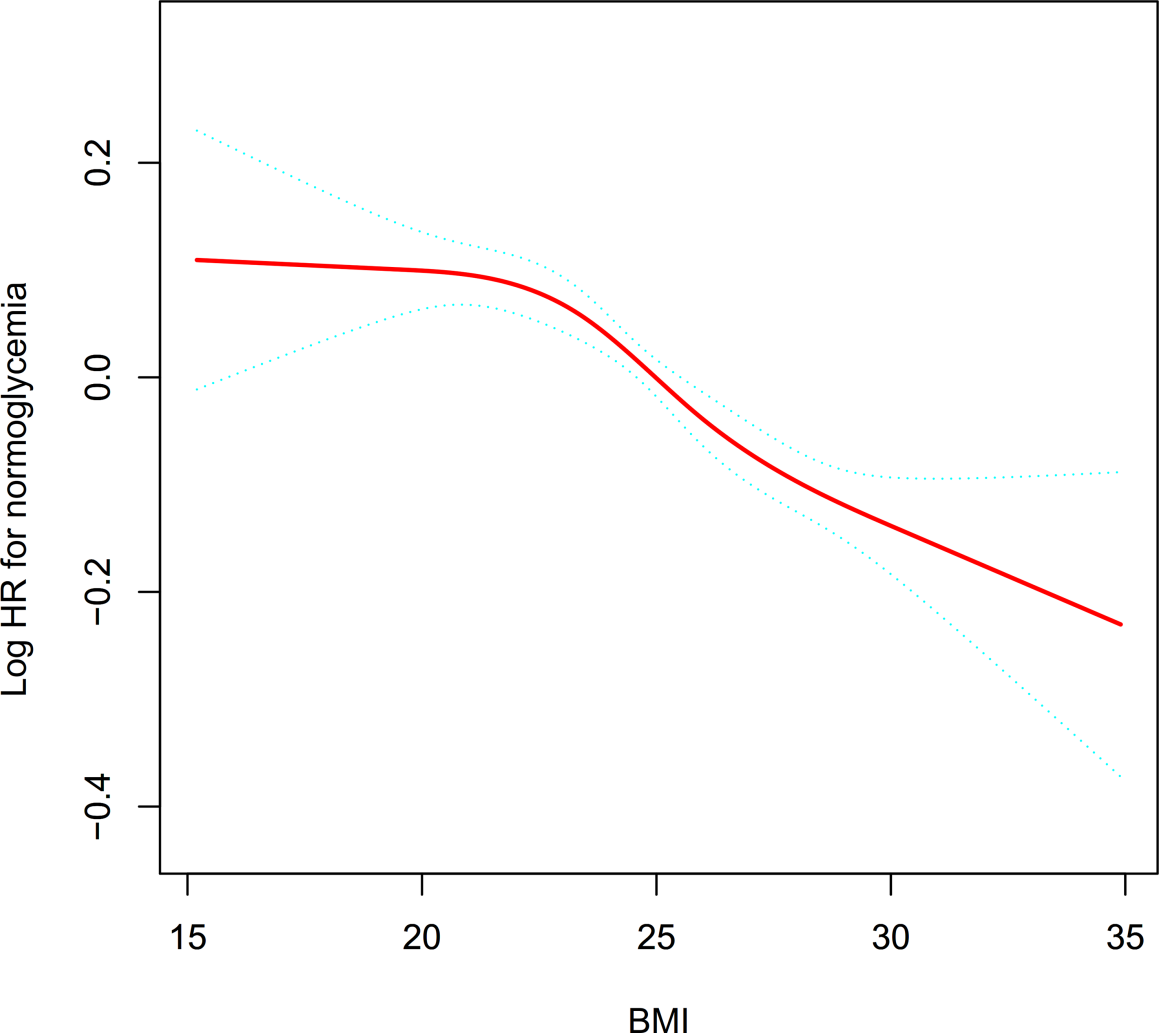
The nonlinear relationship between BMI and reversion to normoglycemia in patients with IFG. The result showed that the relationship between BMI and reversion to normoglycemia from IFG was nonlinear, with the inflection point of BMI being 21.7kg/m^2^.

Using a Cox proportional hazards regression model with cubic spline functions, we found that the correlation between BMI and the probability of reversal to normoglycemia in patients with IFG was nonlinear (**Figure 6**). Additionally, using a standard binary two-piecewise Cox proportional-hazards regression model to fit the data, we chose the model that best fit the data using the log-likelihood ratio test (**Table 7**). Less than 0.05 was the P-value for the log-likelihood ratio test. By using the recursive technique, we first established the 21.7 kg/m^2^ as the BMI inflection point. Next, we utilized a two-piecewise Cox proportional hazards regression model to get the HR and CI for either side of the inflection point. Before the inflection point, the HR was 1.000 (95% CI: 0.978, 1.022, P=0.979), which was not statistically significant, and after the inflection point, the HR was 0.972 (95% CI: 0.964-0.980).

**Table 7:** The result of two-piecewise linear regression model.

Outcome: reversion to normoglycemia	HR, 95%CI	P-value
Fitting model by standard Cox regression	0.977 (0.971, 0.984)	<0.001
Fitting model by two-piecewise Cox regression		
Inflection points of BMI(Kg/m^2^)	21.7	
**< 21.7** kg/m^2^	1.000 (0.978, 1.022)	0.979
**≥21.7** kg/m^2^	0.972 (0.964, 0.980)	<0.001
P for log-likelihood ratio test	0.035	

### Results of subgroup analysis

In all prespecified or exploratory subgroups assessed (**Table 8**), sex, age, smoking status, SBP, and alcohol consumption did not modify the relationship between BMI and reversion to normoglycemia from IFG. That is, there was no significant interaction between these factors and BMI (P > 0.05 for interaction).

**Table 8:** Stratified associations between BMI and reversion to normoglycemia in patients with IFG by age, sex, SBP, DBP, smoking status, and drinking status.

Characteristic	No of participants	HR (95%CI)	P value	P for interaction
Age(years)				0.2341
<30	1584	0.996 (0.980, 1.012)	0.6072	
30 to <40	6246	0.993 (0.983, 1.003)	0.1636	
40 to <50	5679	0.967 (0.954, 0.979)	<0.0001	
50 to <60	6015	0.967 (0.953, 0.982)	<0.0001	
60 to <70	4293	0.960 (0.943, 0.977)	<0.0001	
≥70	2057	0.972 (0.949, 0.996)	0.0234	
Sex				0.3776
Male	17168	0.974 (0.966, 0.982)	<0.0001	
Female	8706	0.983 (0.973, 0.992)	0.0004	
SBP (mmHg)				0.1509
<140	20404	0.976 (0.969, 0.983)	<0.0001	
≥140	5470	0.984 (0.970, 0.999)	0.0316	
Drinking status				0.732
Current drinker	965	0.961 (0.927, 0.996)	0.0309	
Ever drinker	3899	0.972 (0.957, 0.987)	0.0004	
Never	21010	0.979 (0.972, 0.986)	<0.0001	
Smoking status				0.1727
Current smoker	5902	0.980 (0.967, 0.993)	0.0034	
Ever smoker	1130	0.950 (0.923, 0.977)	0.0004	
Never	18842	0.978 (0.971, 0.985)	<0.0001	

Above model adjusted for age, sex, SBP, DBP ALT, AST, BUN, Scr, TG, LDL-c, HDL-c, family history of diabetes, drinking status, and smoking status.

In each case, the model is not adjusted for the stratification variable.

HR, Hazard ratios; CI, confidence, Ref, reference.

## Discussion

This retrospective cohort study was designed to examine the link between BMI and reversion to normoglycemia in patients with IFG. We found that the increase in BMI was related to a significantly decreased probability of regression to normoglycemia. A significantly lower probability of reversal from IFG to normoglycemia in overweight and obese patients compared with participants with normal BMI. In addition, a threshold effect curve was discovered, and on both sides of the inflection point, different associations between BMI and reversion to normoglycemia can be identified.

A prospective cohort study of 491 participants showed that during a median follow-up of 2.5 years, 22.6% of participants with prediabetes returned to normoglycemia (41). Results from another study found that one year after the start of follow-up, 54% of participants with prediabetes had returned to normoglycemia, and 6% had developed diabetes (17). Besides, in another cohort study from China, including 14,231 Chinese adults, 44.9% of patients with prediabetes reverted to normoglycemia within 2 years (42). Our study showed that 45.82% of IFG patients returned to normoglycaemia during the 5-year follow-up period. Variations in the rate of reversion to normoglycemia from prediabetes between studies may be attributable to changes in participant age, follow-up length, and ethnicity. It is important to note that all studies have confirmed that a sizable fraction of persons with prediabetes reverts to normoglycemia. Therefore, finding the contributing factors for the reversion to normoglycemia from prediabetes is particularly important for the prevention of diabetes and its complications.

There have been many findings in the past suggesting that elevated BMI is associated with a higher risk of developing diabetes (43–46). In people with prediabetes, increased BMI was also positively associated with the risk of developing diabetes (19). In addition, weight gain is also a high-risk risk factor for prediabetes (21). Several studies have demonstrated that BMI follows a positive dose-response relationship with the risk of prediabetes (47–49). Therefore, we hypothesized that a reduction in BMI may be associated with an increased probability of regression to normoglycemia from prediabetes. Unfortunately, there are few reports on the relationship between them. A study found that a 1 kg/m^2^ increase in delta-BMI (BMI follow-up baseline) was related to a 28% decrease in the odds ratio (OR) for regression to normoglycemia in subjects with prediabetes. Results from another study suggested that Each 5.3 kg/m^2^ increase in BMI was associated with a 6% reduction in the probability of returning to normoglycemia in patients with prediabetes during a median follow-up of 2.5 years (HR=0.94, 95% CI: 0.91–0.98) (41). Our study complemented the existing literature, which supported the hypothesis that elevated BMI was associated with a reduced probability of reversal to normoglycemia in patients with prediabetes. Compared with other studies, the independent variables in our study used both BMI as a categorical variable and a continuous variable of BMI to explore its relationship with reversion to normoglycemia from prediabetes, which reduced the loss of information and quantified their relationship. In addition, the covariates adjusted for our study were different from those of the previous studies. We adjusted more parameters, including drinking status, smoking status, ALT, AST, and LDL-c. Evidence showed that those parameters were associated with the development of diabetes (50–53). Meanwhile, the sensitivity analysis found that this relationship still exists among participants with TG < 1.7 mmol/l, no family history of diabetes, and never alcohol consumption. Furthermore, we applied a competing risk multivariate Cox regression analysis model, and the results were consistent with those of a multivariate Cox proportional hazards regression model. The results mentioned above have confirmed the relationship stability between BMI and reversion to normoglycemia in patients with IFG. This finding provides a reference for the clinical intervention of BMI levels to increase the probability of reversal to normoglycemia in patients with IFG. It is worth noting that this study addressing nonlinearity is a great improvement compared to previous studies.

The mechanism underlying the inverse relationship between BMI and reversion to normoglycemia in patients with IFG remains unclear, but it may be associated with insulin resistance(IR). Research has confirmed that IR plays a crucial role in the regression and progression of prediabetes (27). In addition, evidence shows that BMI is independently positively related to indices of IR and negatively related to β-cell function adjusted for IR (54).

Furthermore, this study utilized a model based on a two-piecewise Cox proportional hazards regression to shed light on the nonlinear connections. The findings demonstrated a nonlinear link and threshold effect between BMI and reversion to normoglycemia from IFG. The inflection point of BMI was 21.7kg/m^2^ after adjusting for confounders. There was no significant association between elevated BMI and reversal of normoglycemia in IFG patients when BMI was below 21.7 kg/m^2^. However, when BMI was greater than 21.7kg/m^2^, the probability of reversal to normoglycemia decreased by 2.8% for every 1kg/m^2^ increase in BMI. That is to say, as the BMI of patients with IFG decreases, the probability of reversal to normoglycemia will gradually increase, but when it drops to about 21.7kg/m^2^, the probability of reversal to normoglycemia will not continue to increase and remain stable. The possible reason for the non-linear association between BMI and reversion to normoglycemia in patients with prediabetes is that the risk of IR decreases as BMI decreases, but the risk of IR does not continue to decrease when BMI decreases to a certain extent (54). In addition, studies have confirmed that skeletal muscle plays an important role in glucose metabolism. It is one of the major components of insulin-mediated glucose metabolism. Maintaining and increasing skeletal muscle mass can improve IR (55). An excessively low BMI is often accompanied by reduced skeletal muscle mass, reduced insulin sensitivity and abnormalities in glucose and fatty acid metabolism (56, 57). Therefore, the reason that the probability of reversal to normoglycemia does not continue to increase with a decrease in BMI to 21.7 kg/m^2^ may be that a decrease in skeletal muscle mass offsets the benefits of a continued decrease in BMI. Excellent clinical value can be derived from the finding that BMI and reversion to normoglycemia in patients with prediabetes have a curvilinear relationship. It promotes clinical consultation and offers a reference for decision-making that is optimized for diabetes prevention. The population with prediabetes is at much higher risk not only for T2DM but also for cardiovascular disease and all-cause mortality (58, 59). Previous research has demonstrated that even a brief recovery to normoglycemia is associated with a significantly decreased risk of developing T2DM in patients with prediabetes (14). As a result, prediabetes should be treated, and the goal should be regression to normoglycemia rather than only preventing the potential impacts of prediabetes and lowering the likelihood of advancement to T2DM. Lifestyle interventions including diet and exercise have been demonstrated to be useful in the prevention and treatment of prediabetes and T2DM (60). Our study establishes a BMI threshold for the reversion to normoglycemia in Chinese persons with IFG. That is, controlling BMI around 21.7 kg/m^2^ through dietary interventions and lifestyle changes may significantly increase the probability of reversion to normoglycemia.

This study has several strengths worth mentioning. (i) The non-linear association between BMI and recovery from prediabetes to normoglycemia, and the identification of inflection points, are important findings of this study. (iii) To deal with the missing data, we used a multiple imputation approach. This approach allows for maximum statistical power while minimizing bias due to missing covariate information. (iv) A series of sensitivity analyses were conducted to ensure the reliability of the findings. In addition, we performed a multivariate Cox proportional hazards regression model of competing risks, taking into account prediabetes development to diabetes as the competing risk for reversion to normoglycemia event.

The following are some possible limitations of this study. First, as the participants in the study were all Chinese, more investigation is needed to determine the association between BMI and return to normoglycemia in people with prediabetes with different genetic backgrounds. Second, IFG does not fully define prediabetes, however, measuring 2-hour oral glucose tolerance tests and HbA1C is difficult for such a large study cohort. In the future we will conduct our study or collaborate with others as we try to collect information on 2-hour oral glucose tolerance tests and HbA1C levels. Third, this study is based on a secondary analysis of published data; therefore, it is impossible to adjust variables not included in the original dataset, such as insulin concentration and waist circumference. However, we calculated the E-value to quantify the potential impact of unmeasured confounders and found that unmeasured confounders were unlikely to explain the results. In addition, this *post hoc* observational investigation established an association inference between BMI and regression of normoglycemia in patients with IFG rather than a causal one. Finally, the BMI and other parameters were only evaluated at baseline in the current study, and their variations over time were not considered. In the future, we can also think about structuring our studies or working with other researchers to get as many data points as we can, such as details on how BMI changes over the course of patient follow-up.

## Conclusion

This study showed that BMI was independently associated with regression to normoglycemia in Chinese adults with IFG and that there was a specific non-linear relationship and threshold effect between them. There was a significant negative correlation between BMI and the likelihood of returning to normoglycemia from IFG when BMI was greater than 21.7 kg/m^2^. Minimizing BMI to 21.7 kg/m^2^ in patients with IFG may significantly increase the probability of returning to normoglycemia.

## Data availability statement

The original contributions presented in the study are included in the article/**Supplementary Materials**, further inquiries can be directed to the corresponding author/s.

## Ethics statement

The studies involving human participants were reviewed and approved by The rich healthcare group review board. The ethics committee waived the requirement of written informed consent for participation.

## Author contributions

YH, and ZH contributed to the study design and drafted the manuscript. HH and YH are responsible for statistical analysis, research, and interpretation of the data. They are responsible for the data’s integrity and the data analysis’s accuracy. HH and DL contributed to the discussion and reviewed the manuscript. All authors read and approved the final manuscript.
