# Supplementary figures and images for: Association between body mass index and reversion to normoglycemia from impaired fasting glucose among Chinese adults: a 5-year cohort study
Source: Front Endocrinol (Lausanne). 2023 Apr 18;14:1111791. doi: 10.3389/fendo.2023.1111791 (PMC10151769; doi:10.3389/fendo.2023.1111791)

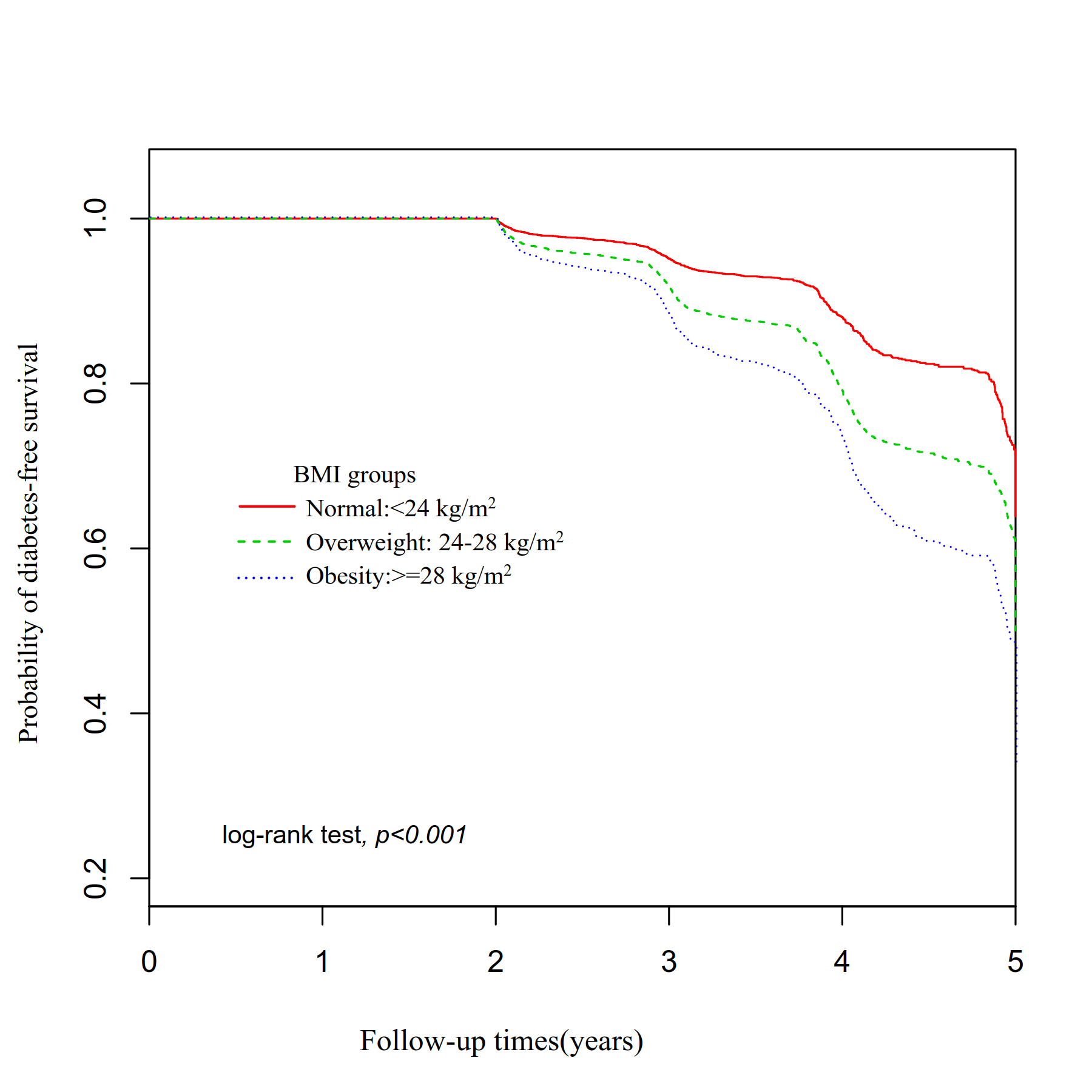

Supplement: Supplementary file 1 [file Image_1.tif]

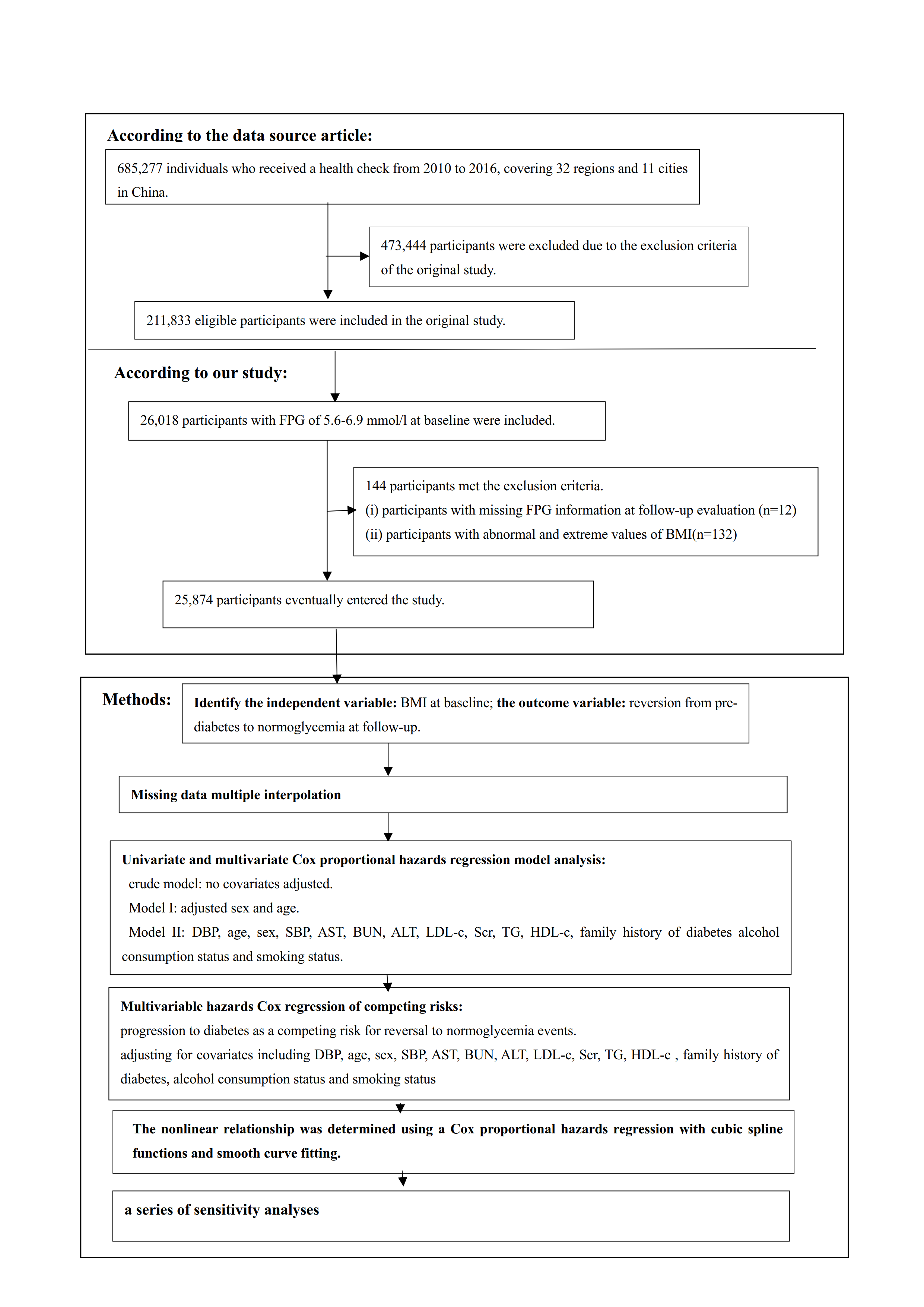

Supplement: Supplementary file 2 [file Image_2.tif]
