# Supplementary material for: Association between body mass index and reversion to normoglycemia from impaired fasting glucose among Chinese adults: a 5-year cohort study
Source: Front Endocrinol (Lausanne). 2023 Apr 18;14:1111791. doi: 10.3389/fendo.2023.1111791 (PMC10151769; doi:10.3389/fendo.2023.1111791)
Supplement: Supplementary file 4 [file Table_2.docx]

**Association between body mass index and reversion to normoglycemia from impaired fasting glucose among Chinese adults: a cohort study**

**Running title:** **BMI and reversion to normoglycemia**

**Yong Han^1#^,** **Haofei Hu^2#^, Zhiqiang Huang^1*^, Dehong Liu^1*^**

^1^Department of Emergency, Shenzhen Second People's Hospital, Shenzhen 518000, Guangdong Province, China

^2^Department of Nephrology, Shenzhen Second People's Hospital, Shenzhen 518000, Guangdong Province, China

**^#^Yong Han, and Haofei Hu have contributed equally to this work.**

***Corresponding author**

Zhiqiang Huang

Department of Emergency,

Shenzhen Second People's Hospital

No.3002 Sungang Road, Futian District,

Shenzhen 518000,

Guangdong Province,

China

E-mail: [huangzhiqiang444@163.com](mailto:huangzhiqiang444@163.com)

***Corresponding author**

Dehong Liu

Department of Emergency, Shenzhen Second People's Hospital

No.3002 Sungang Road, Futian District,

Shenzhen 518035,

Guangdong Province,

China

Orcid id:[0000-0001-9615-5119](https://orcid.org/0000-0001-9615-5119)

E-mail: dhliu_emergency@163.com

Table S1 Collinearity screening

|  | Step 1 | Step 2 |
| --- | --- | --- |
| BMI (kg/m^2^) | 1.3 | 1.3 |
| Age(years) | 1.4 | 1.4 |
| Sex | 1.9 | 1.9 |
| SBP (mmHg) | 1.9 | 1.9 |
| DBP (mmHg) | 1.8 | 1.8 |
| TC (mmol/L) | 6.4 | NA |
| TG (mmol/L) | 1.9 | 1.2 |
| HDL-c(mmol/L) | 1.4 | 1.2 |
| LDL-c(mmol/L) | 5.3 | 1.1 |
| ALT(U/L) | 3.5 | 3.5 |
| ALT(U/L) | 3.2 | 3.2 |
| BUN (mmol/L) | 1.2 | 1.2 |
| Scr (μmol/L) | 1.8 | 1.8 |
| Drinking status | 1.1 | 1.1 |
| Family history of diabetes | 1 | 1 |
| Smoking status | 1.2 | 1.2 |

Variables excluded from collinearity screening: TC

Abbreviations: DBP, diastolic blood pressure; BMI, body mass index; TC, total cholesterol, SBP, systolic blood pressure; TG triglyceride, BMI, body mass index; AST aspartate aminotransferase; LDL-c, low-density lipid cholesterol; ALT, alanine aminotransferase; BUN, blood urea nitrogen; HDL-c, high-density lipoprotein cholesterol; Scr, serum creatinine.

**Table S2 Baseline characteristics according to reversal and progression status of patients with prediabetes**

| Outcomes | Persistent prediabetes | reversion to normoglycemia | Progression to diabetes | P-value |
| --- | --- | --- | --- | --- |
| participants | 11299(43.67%) | 11856(45.82%) | 2719(10.51%) |  |
| Age(years) | 51.25 ± 13.35 | 45.50 ± 13.57 | 55.58 ± 12.47 | <0.001 |
| Height(cm) | 166.63 ± 8.27 | 166.74 ± 8.39 | 166.82 ± 8.34 | 0.425 |
| Weight(kg) | 69.84 ± 11.71 | 67.37 ± 11.91 | 72.88 ± 11.98 | <0.001 |
| BMI (kg/m^2^) | 25.06 ± 3.16 | 24.13 ± 3.24 | 26.09 ± 3.10 | <0.001 |
| SBP (mmHg) | 129.05 ± 17.65 | 124.08 ± 16.67 | 132.33 ± 18.44 | <0.001 |
| DBP (mmHg) | 79.51 ± 11.19 | 76.73 ± 10.74 | 80.80 ± 11.50 | <0.001 |
| TC (mmol/L) | 5.03 ± 0.96 | 4.89 ± 0.95 | 5.09 ± 0.96 | <0.001 |
| TG (mmol/L) | 1.86 ± 1.43 | 1.63 ± 1.41 | 2.18 ± 1.59 | <0.001 |
| HDL-c(mmol/L) | 1.33 ± 0.30 | 1.34 ± 0.30 | 1.29 ± 0.34 | <0.001 |
| LDL-c(mmol/L) | 2.91 ± 0.72 | 2.84 ± 0.72 | 2.92 ± 0.72 | <0.001 |
| ALT(U/L) | 23.00 (16.00-33.90) | 20.20 (14.10-31.00) | 26.60 (18.60-41.00) | <0.001 |
| AST(U/L) | 26.89 ± 12.10 | 25.25 ± 11.24 | 29.50 ± 13.57 | <0.001 |
| BUN (mmol/L) | 5.04 ± 1.24 | 4.92 ± 1.25 | 5.06 ± 1.26 | <0.001 |
| Scr (μmol/L) | 73.52 ± 15.72 | 72.00 ± 16.32 | 73.13 ± 15.94 | <0.001 |
| Sex |  |  |  | <0.001 |
| Male | 7739 (68.49%) | 7435 (62.71%) | 1994 (73.34%) |  |
| Female | 3560 (31.51%) | 4421 (37.29%) | 725 (26.66%) |  |
| Smoking status |  |  |  | <0.001 |
| Current smoker | 2669 (23.62%) | 2392 (20.18%) | 841 (30.93%) |  |
| Ever smoker | 502 (4.44%) | 482 (4.07%) | 146 (5.37%) |  |
| Never | 8128 (71.94%) | 8982 (75.76%) | 1732 (63.70%) |  |
| Drinking status |  |  |  | <0.001 |
| Current drinker | 468 (4.14%) | 349 (2.94%) | 148 (5.44%) |  |
| Ever drinker | 1730 (15.31%) | 1746 (14.73%) | 423 (15.56%) |  |
| Never | 9101 (80.55%) | 9761 (82.33%) | 2148 (79.00%) |  |
| Family history of diabetes | |  |  | <0.001 |
| No | 11037 (97.68%) | 11599 (97.83%) | 2608 (95.92%) |  |
| Yes | 262 (2.32%) | 257 (2.17%) | 111 (4.08%) |  |

Continuous variables were summarized as mean (SD) or medians (quartile interval); categorical variables were displayed as percentage (%)

Abbreviations: DBP, diastolic blood pressure; BMI, body mass index; TC, total cholesterol, SBP, systolic blood pressure; TG triglyceride, BMI, body mass index; AST aspartate aminotransferase; LDL-c, low-density lipid cholesterol; ALT, alanine aminotransferase; BUN, blood urea nitrogen; HDL-c, high-density lipoprotein cholesterol; Scr, serum creatinine.
